# Antigenic and genetic characterization of influenza viruses circulating in Bulgaria during the 2015/2016 season

**DOI:** 10.1016/j.meegid.2017.01.027

**Published:** 2017-04

**Authors:** Neli Korsun, Svetla Angelova, Viki Gregory, Rodney Daniels, Irina Georgieva, John McCauley

**Affiliations:** a National Laboratory “Influenza and ARD”, National Centre of Infectious and Parasitic Diseases, 44A Stoletov Blvd, 1233 Sofia, Bulgaria; b WHO Collaborating Centre for Reference and Research on Influenza, Crick Worldwide Influenza Centre, The Francis Crick Institute, 1, Midland Road, London NW1 1AT, United Kingdom

**Keywords:** Influenza virus, Antigenic and genetic characterization, Amino acid substitution

## Abstract

Influenza virological surveillance is an essential tool for early detection of novel genetic variants of epidemiologic and clinical significance. The *aim* of this study was to determine the antigenic and molecular characteristics of influenza viruses circulating in Bulgaria during the 2015/2016 season. The season was characterized by dominant circulation of A(H1N1)pdm09 viruses, accounting for 66% of detected influenza viruses, followed by B/Victoria-lineage viruses (24%) and A(H3N2) viruses (10%). All sequenced influenza A(H1N1)pdm09, A(H3N2) and B/Victoria-lineage viruses belonged to the 6B.1, 3C.2a and 1A genetic groups, respectively. Amino acid analysis of 57 A(H1N1)pdm09 isolates revealed the presence of 16 changes in hemagglutinin (HA) compared to the vaccine virus, five of which occurred in four antigenic sites, together with 16 changes in neuraminidase (NA) and a number of substitutions in proteins MP, NP, NS and PB2. Despite the many amino acid substitutions, A(H1N1)pdm09 viruses remained antigenically closely related to A/California/7/2009 vaccine virus. Bulgarian A(H3N2) strains (subclade 3C.2a) showed changes at 11 HA positions four of which were located in antigenic sites A and B, together with 6 positions in NA, compared to the subclade 3C.3a vaccine virus. They contained unique HA1 substitutions N171K, S312R and HA2 substitutions I77V and G155E compared to Bulgarian 3C.2a viruses of the previous season. All 20 B/Victoria-lineage viruses sequenced harboured two substitutions in the antigenic 120-loop region of HA, and 5 changes in NA, compared to the B/Brisbane/60/2008 vaccine virus. The results of this study reaffirm the continuous genetic variability of circulating seasonal influenza viruses and the need for continued systematic antigenic and molecular surveillance.

## Highlights

• 6B.1, 3C.2a and 1A were the most prevalent genetic subclades of A(H1N1)pdm09, A(H3N2) and B/Victoria viruses, respectively.
• Differences in antigenic sites, RBS and *N*-glycosylation motifs in HA and NA between epidemic and vaccine viruses were found.
• Amino acid substitutions in internal proteins M, NP, NS1 and PB2 were identified.
• All influenza A viruses were resistant to M2 blockers but both A/B viruses were susceptible to oseltamivir and zanamivir.

## 1 Introduction

Of the many viral respiratory infections in humans, influenza has the greatest clinical and epidemiologic importance. Each year 600 million influenza cases occur worldwide, with 3 million having severe disease and 250,000–500,000 resulting in death (WHO, 2014). Periodically, at intervals of 10 to 40 years since 1890, influenza type A viruses cause pandemics resulting from the emergence of a radically new subtype/variant influenza virus against which the human population has little or no preexisting immunity — a process called antigenic shift. The last influenza pandemic in 2009/2010 was caused by A(H1N1)pdm09 virus containing a complex combination of gene segments from swine, avian and human influenza viruses (Neumann et al., 2009). This virus completely replaced former seasonal A(H1N1) viruses and continues to circulate worldwide as a seasonal influenza virus together with A(H3N2) and type B viruses.

Influenza vaccines were developed to reduce the substantial influenza-associated morbidity and mortality but their effectiveness declines over time due to emerging genetic and associated antigenic differences between vaccine and circulating viruses. Influenza viruses are one of the most variable and rapidly evolving human viruses because of their high mutation rate, rapid replication, segmented genome (which facilitates the reassortment of genes between different influenza viruses) and zoonotic events for type A viruses. Evolution of influenza viruses proceeds by continuous replacement of genetic groups with new ones leading to increases in the antigenic distances from the current vaccine viruses.

The influenza virus surface glycoproteins, HA and NA are subjected to the strongest pressure by the host immune system resulting in a gradual accumulation of amino acid changes and altered antigenicity. This process, known as antigenic drift, enables circulating viruses to evade host immune responses leading to recurrent seasonal epidemics and reduction of vaccine effectiveness, necessitating updates of vaccine composition. Distinct antigenic sites in A/H1 (Sa, Sb, Ca 1/2, Cb), A/*H*3 (A-E) and in type B viruses (120 loop, 150 loop, 160 loop, 190 helix) located on the globular head of the HA1 subunit are targets of neutralizing antibodies (Wiley and Skehel, 1987, Wilson and Cox, 1990). Amino acid substitutions within epitopes and the attachment of *N*-glycans to the globular head region of HA, shielding antigenic epitopes, can reduce the recognition of virus by neutralizing antibodies and thus resulting in the escape from pre-existing immunity (Skehel et al., 1984).

The World Health Organization (WHO) encourages National Influenza Centers (NICs) to conduct ongoing influenza virologic surveillance, to monitor spread of viruses and their continuous evolution to inform twice yearly recommendations on vaccine composition and assessing other risks associated with circulating influenza viruses. Combining data from phylogenetic and molecular analyses of influenza viruses is essential to detect virus variants that have undergone antigenic drift, variants with enhanced virulence or variants reduced sensitivity to antivirals. Such combined genetic, antigenic and phenotypic analyses provide improvements in the process of vaccine virus selection and inform patient treatment regimens. The *aim* of the present study was to analyse influenza virus circulation in Bulgaria during the 2015/2016 season and determine the genetic and antigenic characteristics of the detected viruses related to amino acid changes at antigenic, *N*-glycosylation and functionally significant sites of HA and NA.

## 2 Material and methods

### 2.1 Study population and specimen collection

From October 2015 to May 2016, patients, who were ambulatory treated or hospitalized either for influenza like illness (ILI) or acute respiratory illness (ARI), were enrolled in different regions of the country. Combined nasal and pharyngeal specimens from the enrolled patients were collected with the help of commercial polyester collection swabs (Deltalab, Spain). Swabs were stored at 4 °C for up to 72 h before shipment to the NIC. Specimens were processed immediately or stored at − 80 °C before testing.

### 2.2 Extraction of nucleic acids and real time RT-PCR

Virus RNAs were extracted automatically from the respiratory specimens using a commercial ExiPrep Dx Viral DNA/RNA kit (Bioneer, Korea) in accordance with the manufacturer's instructions. Detection and typing/subtyping of influenza viruses were carried out by a Real Time RT-PCR method with the use of a kit — SuperScript III Platinum ® One-Step qRT-PCR System (Invitrogen, USA). All samples were first tested for the presence of influenza A and B viruses. Those that were positive for influenza A were subsequently screened for A(H1N1)pdm09 and A(H3N2). The genetic lineage of detected influenza B viruses was also determined by Real Time RT-PCR. Primers, probes and positive controls were provided by WHO-CC, Atlanta. Amplification was performed with a Chromo 4 thermal cycler (Bio-Rad) in accordance with the protocol of WHO-CC, Atlanta (reverse transcription at 50 °C for 30 min, Taq inhibitor inactivation at 95 °C for 2 min, followed by 45 cycles of denaturation at 95 °C for 15 s and annealing/amplification at 55 °C for 30 s). A C_t_ value < 38 was regarded as positive.

### 2.3 Virus isolation and antigenic characterization

All Real Time RT-PCR positive clinical specimens with C_t_ values < 28 were inoculated onto Madin Darby canine kidney (MDCK) and MDCK-SIAT1 (that express increased levels of α2,6-sialyltransferase, Matrosovich et al., 2003) cell cultures. Cultures were incubated at 35 °C in a 5% CO_2_ atmosphere and observed daily for 7 days for evidence of cytopathology. The presence of virus in culture was confirmed by haemagglutination assay following standard protocols using a 1% suspension of guinea pig red blood cells. Antigenic characterization of isolates was performed by the haemagglutination inhibition (HI) assay, in accordance with the WHO Manual, using vaccine viruses/antigens and their corresponding antisera provided by the WHO-CCs in London and Atlanta (WHO, 2011). More detailed HI assay of representative Bulgarian influenza isolates with panels of reference viruses and antisera were performed at the WHO-CCs in London and Atlanta.

### 2.4 Genetic characterization

Full-genome or HA and NA gene sequences of influenza viruses detected in Bulgaria during the 2015/2016 season were determined at WHO-CC, London. Full-genome sequencing was carried out at WHO-CC, Atlanta. Sequences have been deposited in the Global Initiative on Sharing All Influenza Data (GISAID) database (http://www.gisaid.org) with sequences of all but three viruses being derived from virus isolates that were characterized antigenically. For phylogenetic analyses all sequences, including those of reference viruses whose genetic group identities were known and viruses representing different countries of Europe during the 2015/2016 season, were retrieved from GISAID. Phylogenies for HA and NA genes were constructed using the maximum likelihood method within Molecular Evolutionary Genetics Analysis software (MEGA, version 6.0; http://www.megasoftware.net/). Best nucleotide substitution models were used: the Hasegawa-Kishino-Yano model with a gamma distribution (HKY + G) for HA; and the Tamura 3-parameter model with gamma distribution (T92 + G) for NA. Reliability of the tree topology was assessed by bootstrap analysis with 1000 replications. HA amino acid numbering was applied after removing the signal peptide. Amino acid identity was calculated using flusurver (http://flusurver.bii.a-star.edu.sg).

### 2.5 Prediction of N-glycosylation motifs

Putative N-glycosylation motifs in the HA and NA were predicted using the NetNGlyc 1.0 web Server (http://www.cbs.dtu.dk/services/ NetNGlyc) to identify sequence motifs N–X–S/T (sequon), where X can be any amino acid except proline.

### 2.6 Antiviral susceptibility surveillance

Screening of A(H1N1)pdm09 viruses for the presence of point mutations conferring H275Y oseltamivir resistance was carried out using a Real Time RT-PCR assay that allowed discrimination of a single nucleotide difference between oseltamivir sensitive and resistant viruses. Two TaqMan probes differing in position 823 of the NA gene were used simultaneously: the first probe contained a cytosine at position 823 and was labeled with VIC (H275), while the second probe contained thymine in the same position and was labeled with FAM (275Y). Primer/probe sequences and protocol were kindly provided by Public Health England (formerly Health Protection Agency, England), London. Reference influenza viruses A/Denmark/524/2009 (sensitive, H275) and A/Denmark/528/2009 (resistant, 275Y) provided by WHO-CC, London were used as positive controls. A phenotypic analysis (MUNANA test) of influenza virus susceptibility to neuraminidase inhibitors (oseltamivir and zanamivir) was performed at WHO-CC, London.

### 2.7 Statistics

Age and gender of patients, the clinical features of their illness and the incidence of each virus were compared using the Chi square or Fisher's exact tests for categorical variables. *p* values of < 0.05 were considered statistically significant.

## 3 Results

Bulgaria is a country with a total population of approximately 7.2 million people and an ARI surveillance system is used to monitor influenza. It comprises a national sentinel network of general practitioners and pediatricians working in 208 health care facilities situated in all 28 major cities — regional centers covering 5.3% of the population in the country. Primary care physicians report the weekly number of clinical cases of ARI by age group, collect respiratory specimens and send them to the National Reference Laboratory. The Laboratory is recognised as a WHO NIC. It is the only laboratory in the country that conducts research on influenza viruses and performs testing of clinical samples from severely ill patients hospitalized in different regions of the country.

The first influenza detection, an A(H1N1)pdm09 virus, occurred in week 51/2015 and the 2015/2016 influenza season was characterized as being of average duration and moderate intensity but with a lower incidence rate compared to the previous two seasons. The epidemic lasted seven weeks (from week 2 to week 8) and peaked in week 6/2016, slightly later than the 2014/2015 season, with an incidence rate of 158.74 cases per 10,000 people. As in previous years, the ILI and ARI morbidity rate was the highest in young children < 4 years of age, followed by the 5–14 years age group (www.grippe.gateway.bg).

### 3.1 Influenza virus detection

**Fig. 1:**
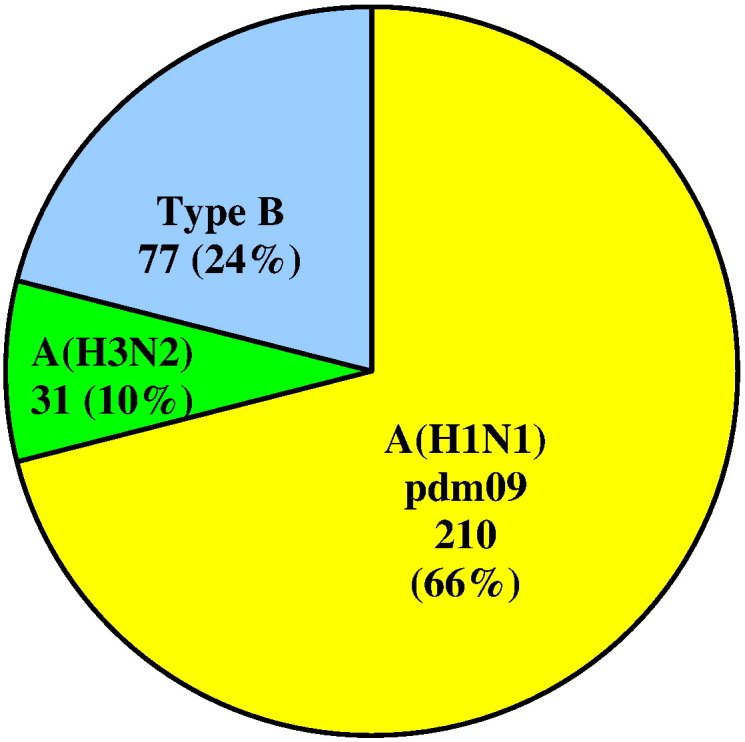
Results of typing/subtyping of influenza viruses detected during the 2015/2016 season.

**Fig. 2:**
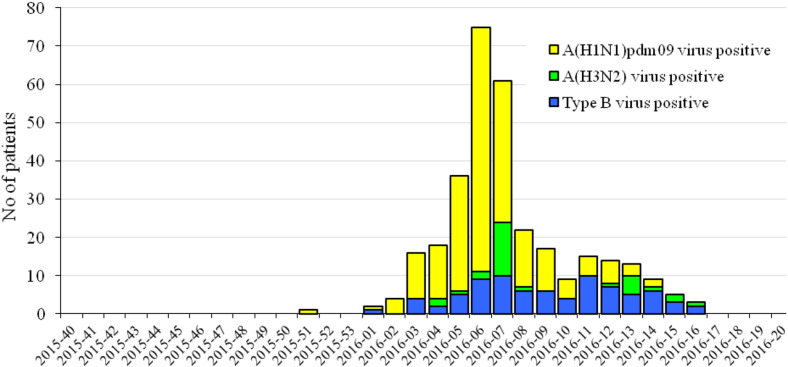
Weekly distribution of influenza virus detections during the 2015/2016 season.

The study population consisted of 1127 patients demonstrating symptoms of ILI or ARI: 218 (19.3%) of these were persons attending outpatient healthcare centers; 909 (80.7%) were inpatients, of which 36 were in intensive care units (ICU). The patients' ages ranged from 25 days to 92 years old (y.o.) (average age 21.7 y.o.) and 51.6% were male. Influenza viruses were detected in 318 (28%) patient samples. Of these, 241 (75.8%) were positive for influenza type A virus and 77 (24.2%) for type B. Among the influenza A viruses, 210 (87%) were A(H1N1)pdm09 and 31 (13%) A(H3N2) viruses (Fig. 1). All detected influenza type B viruses belonged to the Victoria-lineage. In weeks 3–9/2016, A(H1N1)pdm09 viruses dominated representing up to 85% of the detected influenza viruses. Influenza type B positive cases increased from the end of February. The last influenza virus (type B) was detected in week 16/2016 (Fig. 2).

### 3.2 Demographics and clinical characteristics of patients infected with influenza viruses

The average age of influenza virus-positive patients was 21.4 years old (range, 4 months to 87 y.o.) and 53.9% were male. Among outpatients, 17.9% (39/218) were identified as positive for influenza virus infection, rising to 30.7% (279/909) (*p* < 0.05) among hospitalized patients. For patients infected by A(H1N1)pdm09 virus, these proportions were 12.4% (27/218) and 20% (183/909), and 4.6% (10/218) and 7.4% (67/909) for influenza type B virus infected patients, respectively. All age groups were infected by influenza viruses but the highest influenza virus-positivity (31.3%) was found in the 5–14 y.o. age group. Lower percentages of detected viruses were observed in adults aged 30–64 y.o. (29%) and in children 0–4 y.o (27.8%). The predominant A(H1N1)pdm09 viruses were most frequently detected in the 30–64 y.o. (22.7%) and 0–4 y.o. (20.7%) age groups. The proportions of detected A(H1N1)pdm09 viruses in patients 5–14 y.o. and 15–29 y.o. were similar, 13.7% and 15.3% respectively. The highest rate of influenza B detection (13.7%) was in the 5–14 y.o. age group, which showed an equal proportion of A(H1N1)pdm09 virus detection (Table 1).

Influenza viruses were detected in 31% (60/194) of the studied patients diagnosed with pneumonia and in 24% (32/128) of patients with CNS involvement (meningitis, encephalitis, brain edema, encephalopathy). In patients with pneumonia, the detection rates for influenza A(H1N1)pdm09, A(H3N2) and type B viruses were 76.7% (46/60), 5.0% (3/60) and 18.3% (11/60), respectively; and in patients with neurologic complications — 56.3% (18/32), 6.3% (2/32) and 37.4% (12/32), respectively. Among the 36 patients treated in ICUs, A(H1N1)pdm09 and type B viruses were detected in 27.8% (10/36) and 8.3% (3/36), respectively. Of eight deaths, two each were infected A(H1N1)pdm09 or type B viruses.

### 3.3 Virus antigenic characterization

The first five detected influenza viruses (clinical specimens) were sent to WHO-CC, Atlanta for further characterization. In the course of the 2015/2016 season, a total of 171 Real Time RT-PCR positive clinical specimens with C_t_ values < 28 were inoculated onto MDCK and MDCK-SIAT1 cell cultures and 99 of them were successfully cultured after the first or second passage. In total, 81 representative influenza isolates were sent to WHO-CC, London where they were characterized in detail. Fifty-seven Bulgarian A(H1N1)pdm09 isolates, all but two obtained from hospitalized patients, were tested by HI using post-infection ferret antisera raised against the reference viruses presented in Table 2. All 57 viruses were recognised very well by the antiserum raised against the vaccine virus, A/California/7/2009, and by most of the other antisera. The exceptions were antiserum raised against A/Lviv/N6/2009, which recognised 12/57 (21%) viruses at titres 8-fold lower than the homologous titre, and antiserum raised against A/Christchurch/16/2010, which recognised 55/57 (96.5%) viruses at titres ≥ 8-fold lower than the homologous titre. A small number of viruses were recognised by antisera raised against A/Slovenia/2903/2015 (1 virus) and A/Israel/Q-504/2015 (5 viruses) at titres 4-fold reduced compared to the homologous titres of the antisera.

The three Bulgarian A(H3N2) viruses were unable to bind red blood cells and therefore no HI analyses were performed.

Nineteen Bulgarian type B viruses were assessed by HI using post-infection ferret antisera raised against reference viruses indicated in Table 3. All Bulgarian viruses showed low reactivity with antisera raised against the egg-propagated viruses B/Malaysia/2506/2004, B/Brisbane/60/2008, B/Malta/636714/2011, B/Johannesburg/3964/2012 and B/South Australia/81/2012. The test viruses showed 2- to 4-fold reduced reactivity with antisera raised against cell culture-propagated viruses genetically closely related to B/Brisbane/60/2008 — B/Formosa/V2367/2012, B/Ireland/3154/2016 and B/Nordrhein-Westfalen/1/2016.

### 3.4 Genetic characterization

**Fig. 3:**
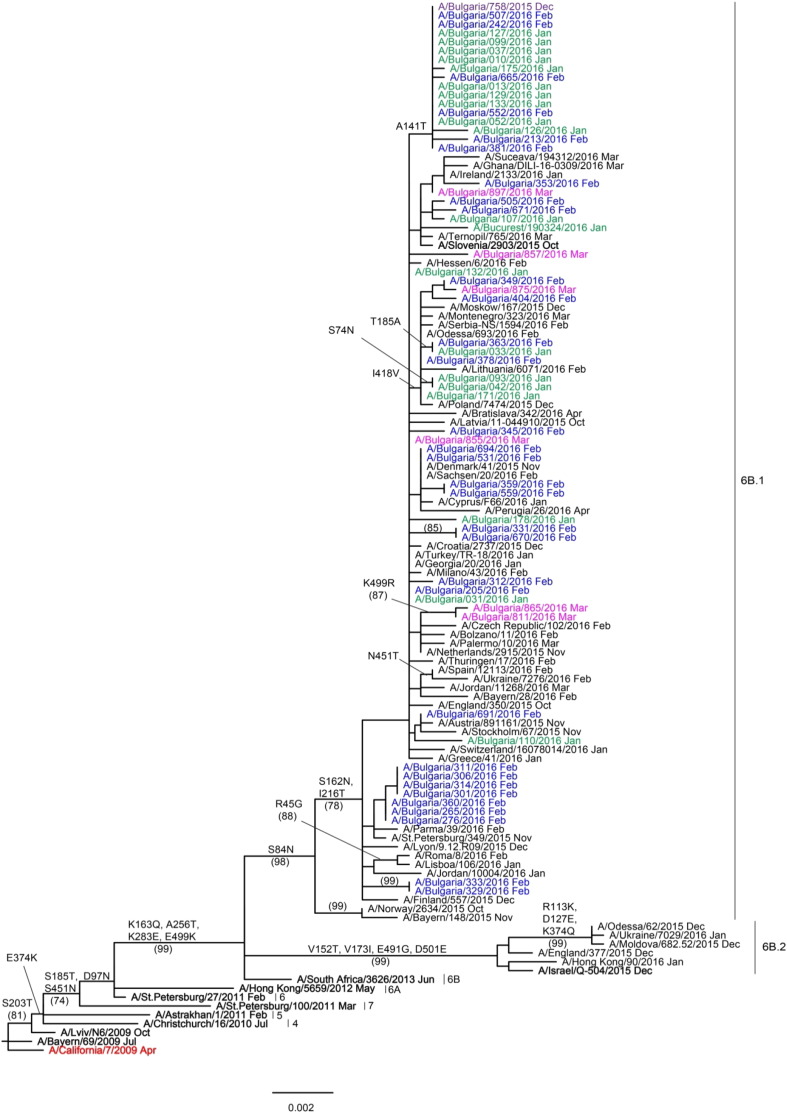
HA gene phylogeny of influenza A(H1N1)pdm09 viruses detected in Bulgaria during the 2015/2016 season. Reference viruses are indicated in bold and vaccine virus A/California/7/2009 in red. Bulgarian viruses detected in December 2015, January, February and March 2016 are indicated in purple, green, blue and pink, respectively. The tree is rooted at A/California/7/2009. Amino acid substitutions defining particular nodes are indicated and bootstrap values > 70% are shown in parentheses.

**Fig. 4:**
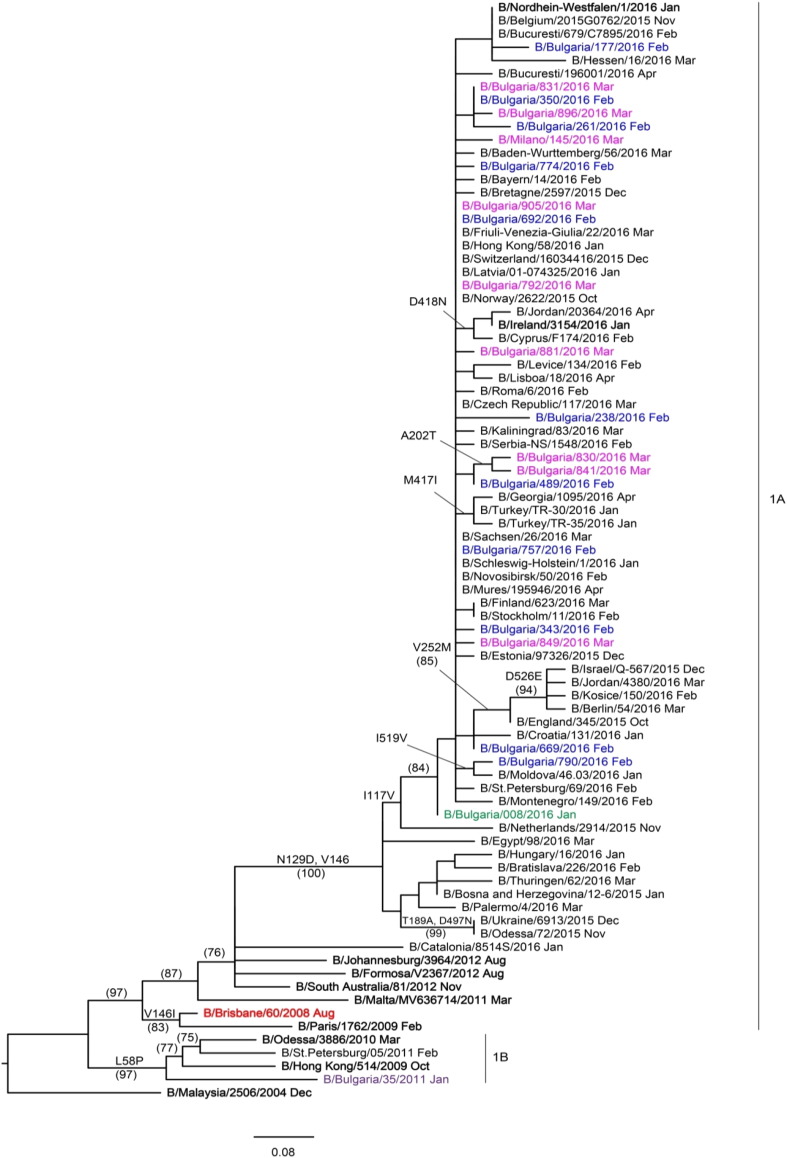
HA gene phylogeny of influenza B/Victoria-lineage viruses detected in Bulgaria during the 2015/2016 season. Reference viruses are indicated in bold and vaccine virus B/Brisbane/60/2008 in red. Bulgarian viruses detected in January, February and March 2016 are indicated in green, blue and pink, respectively. The tree is rooted at B/Malaysia/2506/2004. Amino acid substitutions defining particular nodes are indicated and bootstrap values > 70% are shown in parentheses.

HA and NA gene sequences were recovered from GISAID and phylogenetic trees constructed to determine the genetic relationships of Bulgarian isolates with reference viruses and viruses circulating in other countries in the same period. The 57 HA genes of Bulgarian A(H1N1)pdm09 viruses fell within a new genetic subclade, 6B.1, and clustered with the reference virus A/Slovenia/2903/2015 (Fig. 3), as did the NA genes (results not shown). The three sequenced A(H3N2) viruses belonged to genetic subclade 3C.2a together with the 2016–2017 Northern hemisphere vaccine virus, A/Hong Kong/4801/2014. All 20 B/Victoria-lineage viruses fell into genetic clade 1A represented by the vaccine virus B/Brisbane/60/2008 (Fig. 4). Inter- and intra-lineage reassortments involving HA and NA genes were not detected.

### 3.5 Amino acid sequence analysis

Complete HA and NA amino acid sequences of Bulgarian influenza isolates were compared to those of vaccine viruses to identify substitutions that might impact vaccine effectiveness (Table 4).

#### 3.5.1 A(H1N1)pdm09

HA and NA sequences of the 57 Bulgarian A(H1N1)pdm09 isolates were compared to those of the vaccine virus and representatives of particular genetic groups/subgroups. HA amino acid sequence identity of studied isolates ranged from 97% to 97.4% compared to vaccine virus, A/California/7/2009. All 57 A(H1N1)pdm09 HA sequences contained 11 amino acid changes in HA1 polypeptide and three in HA2: HA1 substitutions P83S, S203T and I321V that were fixed and present in viruses from all genetic groups; substitutions D97N and S185T in HA1 with E47K and S124N in HA2 defining group 6 (A/St. Petersburg/27/2011); HA1 substitutions K163Q, A256T and K283E with E172K in HA2 defining group 6B (A/South Africa/3626/2013) and HA1 substitutions S84N, S162N and I216T defining subclade 6B.1 (exemplified by the reference virus A/Slovenia/2903/2015). In addition, sixteen (28%) viruses carried HA1 A141T substitution and nine (16%) viruses had HA2 I91V substitution. Thirteen viruses carried additional singlе amino acid changes in HA. None of these substitutions were associated with known adaptation to propagation in MDCK culture (Ramadhany et al., 2012). Among the specified amino acid substitutions, five were located in antigenic sites: S203T in site Ca1; A141T in Ca2; S162N and K163Q in Sa and S185T in Sb. Only the S185T substitution falls within a domain defining the receptor binding site (RBS): 190-helix (residues 184–191); 220-loop (218–225); 130-loop (131–135), and in highly conserved residues (Y91, W150, H180 and Y192) (Yang et al., 2010, De Graaf and Fouchier, 2014, Fang et al., 2014, Sriwilaijaroen and Suzuki, 2012). All Bulgarian viruses carried nine conserved potential *N*-glycosylation motifs in HA (HA1 positions 10, 11, 23, 87, 162, 276, 287 and HA2 positions 154 and 213). S162N substitution, specific to subclade 6B.1 viruses, generated a new potential *N*-glycosylation motif within the Sa antigenic site.

NA sequences of the same 57 A(H1N1)pdm09 viruses differed from that of A/California/7/2009 by 15 substitutions: N248D and Y351K in viruses of all genetic groups; N44S, N200S, V241I and N369K in genetic group 6 viruses; I34V, L40I, I321V, N386K and K432E in genetic group 6B viruses; V13I, K264I, N270K and I314M in genetic group 6B.1 viruses. T48A substitution was detected in 7 (12%) Bulgarian viruses and 16 viruses harboured additional NA singlе amino acid changes. The eight functional (R118, D151, R152, R224, E276, R292, R371, Y406) and 11 framework (E119, R156, W178, S179, D/N198, *I*222, E227, H274, E277, N294, E425) residues comprising the NA catalytic site were conserved among all 57 viruses (Colman et al., 1993). All Bulgarian viruses carried eight conserved potential *N*-glycosylation motifs in NA (positions 42, 50, 58, 63, 68, 88, 146 and 235).

Internal protein sequences of Bulgarian A(H1N1)pdm09 viruses were compared to those of A/California/7/2009, reference viruses (A/South Africa/3626/2013, A/Slovenia/2903/2015 and A/New York/61/2015) and epidemic strains detected in Armenia, Ukraine and the Russian Federation, where severe and fatal influenza cases were reported at the beginning of the season (WHO, 2016b, Ilyicheva et al., 2016). In common with other subclade 6B.1 viruses, Bulgarian isolates contained amino acid substitutions in all proteins: Q208K and Y280H in M1; A22T and M105T in NP; D2E, E125D, R224G, A225T, A241T and T268A in NS1; R299K and S453T in PB2. The possible functional importance of these substitutions is not known. As with the vast majority of A(H1N1)pdm09 viruses, M2 proteins carried S31N substitution associated with resistance to M2-channel blockers (amantadine and rimantadine) (Laplante and George, 2014).

#### 3.5.2 A(H3N2)

The similarity of Bulgarian viruses to the Northern hemisphere 2015/2016 vaccine virus A/Switzerland/9715293/2013 (subclade 3C.3a) at the HA amino acid level ranged from 97.5% to 97.6%. All three Bulgarian strains (subclade 3C.2a) showed variations at eight HA1 positions and three HA2 positions compared to the vaccine virus: HA1 — L3I, A128T (resulting in the gain of a *N*-glycosylation motif), N144S (resulting in the loss of *N*-glycosylation motif), F159Y, K160T (resulting in the gain of a *N*-glycosylation motif), N171K, Q311H, S312R, I406V (corresponding to HA2-subunit position 77), G484E (G155E in HA2), D489N (D160N in HA2). Substitutions A128T and N144S are located in antigenic site A while S159Y and K160T are in antigenic site B. The substitutions N144S, F159Y, K160T, N225D and Q311H in HA1, compared to a previous vaccine virus, A/Texas/50/2012, define subclade 3C.2a; Bulgarian viruses fell into a cluster having HA proteins with HA1 N171K and S312R, and HA2 I77V and G155E substitutions. Thirteen potential *N*-glycosylation motifs in HA (HA1 positions 8, 22, 38, 45, 63, 122, 126, 133, 158, 165, 246, and 285, and HA2 position 154) were identified. Compared to A/Switzerland/9715293/2013, loss and gain of potential *N*-glycosylation motifs were observed at HA1 positions144 and 128, respectively, while the HA2 G155E substitution was within a *N*-glycosylation motif (NGT → NET).

The NA of Bulgarian viruses differed from the NA of A/Switzerland/9715293/2013 by 6 amino acid substitutions: S245N (resulting in gain of a *N*-glycosylation motif), S247T, T267K, P339N, I380V and P468H. Nine potential *N*-glycosylation motifs in NA (61, 70, 86, 146, 200, 234, 245, 329, 367) were identified, with two (146 and 367) being located around the enzymatic active site (Fang et al., 2014). Compared to Bulgarian subclade 3C.2a viruses from the previous season, the *N*-glycosylation motif NAT_245–247_ was new. None of the substitutions in HA and NA relate to known MDCK culture-induced substitutions (Lee et al., 2013).

#### 3.5.3 B/Victoria

All 20 Bulgarian B/Victoria-lineage viruses belonged to clade 1A with signature HA1 substitutions, N75K, N165K and S172P, compared to a previous vaccine virus, B/Malaysia/2506/2004. Compared to the current vaccine virus, B/Brisbane/60/2008, HA sequence identity ranged from 99.3% to 99.7%. Two amino acid substitutions were identified in HA, both (I117V and N129D) in the 120-loop (positions 116–137) antigenic site; no amino acid substitutions occurred in the remaining antigenic sites — 150-loop (positions 141–150), 160-loop (positions 162–167) and 190-helix (positions 194–202) (Wang et al., 2008). Seven viruses included in this study carried singlе, unique, substitutions in HA. Twelve putative *N*-glycosylation motifs were identified: HA1 positions 25, 59, 145, 166, 197, 233, 304 and 333, and HA2 positions 145, 171, 184 and 216. *N*-glycosylation motifs 145, 166 and 197 fall in antigenic sites: 150-loop, 160-loop and 190-helix, respectively.

All 20 Bulgarian B/Victoria-lineage viruses carried five NA amino acid substitutions and 11 carried single, unique, substitutions. The catalytic and framework residues in NA were conserved among all 20 viruses studied (Horthongkham et al., 2016). Four putative *N*-glycosylation motifs were identified, at positions 56, 64, 144 and 284, all of which were conserved except in B/Bulgaria/790/2016 which carried N144D substitution.

### 3.6 Antiviral susceptibility surveillance

In the National Laboratory “Influenza and ARD”, all 210 detected A(H1N1)pdm09 viruses were analyzed by real-time RT-PCR with respect to the H275Y oseltamivir resistance substitution — all viruses carried 275H indicative of retained susceptibility. All NA sequences generated were screened for known markers of reduced susceptibility to NA inhibitors (WHO, 2016c) — none were found. Phenotypic testing (by the MUNANA method) for susceptibility to oseltamivir and zanamivir was performed on 57 influenza A(H1N1)pdm09, 3 A(H3N2) and 19 type B viruses: all viruses retained susceptibility to both antiviral drugs.

## 4 Discussion

The antigenic and molecular characteristics of influenza viruses detected in Bulgaria during the 2015/2016 season are described here. In this surveillance period, the total number and percentage (28% vs 26%) of influenza positive cases were slightly higher compared to the previous season when A(H3N2) viruses predominated (Korsun et al., 2015). The study season was characterized by a dominant spread of A(H1N1)pdm09 viruses accounting for 66% of the detected influenza viruses and by low circulation of A(H3N2) viruses (10%). Type B viruses, all belonging to the B/Victoria-lineage, circulated somewhat later and accounted for 24% of influenza viruses detected. Similar proportions of circulating seasonal influenza viruses were observed in most European countries. Cumulative data for the WHO European region showed that within type A, the A(H1N1)pdm09 subtype predominated (91%) over A(H3N2) viruses (9%) and within type B, 91% of the viruses assigned to a lineage were of the Victoria lineage (WHO, 2016d).

There have been year-on-year fluctuations in the distribution and frequency of influenza types/subtypes in Bulgaria since the emergence of A(H1N1)pdm09 viruses in 2009/2010: during the 2010/2011, 2013/2014 and 2015/2016 epidemics, there was clear-cut dominance of A(H1N1)pdm09 viruses; in 2011/2012 and 2014/2015, A(H3N2) viruses prevailed strongly; in 2012/2013 unusually high activity of type B influenza was registered. No A(H1N1)pdm09 viruses were detected in 2011/2012 but they represented 18% of detected viruses in 2011/2012. Vaccine coverage in Bulgaria is very low (approximately 3%) and so is unlikely to significantly affect the circulation of various types/subtypes/lineages of influenza viruses.

Based on patient age, disease burden caused by A(H1N1)pdm09 viruses was greatest in people of active middle age, while type B viruses were most prevalent among children aged 5–14 y.o. in agreement with other studies (Bautista et al., 2010, Beauté et al., 2015). As for previous seasons, in 2015/2016 influenza virus positivity was higher among hospitalized patients (30.7%) compared to outpatients (17.9%) (*p* < 0.05) and a higher percentage of influenza-associated cases of pneumonia (31% vs 14%) and ICU admissions (36% vs 23%) (*p* < 0.05) was found compared to the previous season (Korsun et al., 2015). While in 2014/2015, no fatal cases related to ILI or ARI symptoms were registered, in 2015/2016 clinical materials of eight deceased patients were studied and influenza was detected in four of them. Several European countries — Armenia, Georgia, Russian Federation, Serbia and Ukraine also reported a greater number of severe and fatal cases associated with A(H1N1)pdm09 infection than during the same period of the previous season (WHO, 2016b, Ilyicheva et al., 2016).

During the six seasons since its emergence, the A(H1N1)pdm09 virus has undergone significant genetic change, and eight genetic groups and several subgroups have been defined (WHO, 2016d). Phylogenetic analyses of Bulgarian A(H1N1)pdm09 virus HA and NA sequences revealed variation in circulation of different genetic groups: in 2013/2014 and 2014/2015 genetic group 6B viruses circulated, while in 2015/2016 subclade 6B.1 viruses predominated strongly. The latter viruses have 16 amino acid substitutions each in HA and NA compared to the vaccine virus. Five HA substitutions were located in four antigenic sites; two strain-specific (Sa and Sb) and two common antigenic sites (Ca1 and Ca2) (Table 5). Four of these substitutions were present in all viruses analyzed, while A141T substitution was observed in 28% of the viruses. Substitution S185T was located in the RBS/190-helix (Sriwilaijaroen et al., 2012). It has been reported that substitutions in or near the RBS can influence the antigenic properties of A(H1N1)pdm09 viruses (Koel et al., 2015), and attachment of oligosaccharide chains to antigenic sites has been suggested to facilitate immune evasion. Subclade 6B.1 viruses have an additional *N*-glycosylation motif located within the HA Sa antigenic site. Evolutionary studies of A(H1N1) viruses indicate that the number of *N*-glycosylation motifs in the HA has increased over time while the number in NA is relatively stable. In line with this A(H1N1) viruses from the 1918 pandemic had five potential HA1 *N*-glycosylation motifs and seven in NA, while subclade 6B.1 A(H1N1)pdm09 viruses have seven in HA1 and eight in NA. Despite the above mentioned changes in functionally important sites of HA and NA, no indication of antigenic drift of A(H1N1)pdm09 viruses was observed for Bulgarian isolates, and those worldwide, during the course of the 2015/2016 season, as assessed by use of post-infection ferret sera raised against the A/California/7/2009 vaccine virus in HI assays. However, recent studies have shown that genetic group 6B A(H1N1)pdm09 viruses, including those in subclades 6B.1 and 6B.2, were antigenically distinguishable from the vaccine virus by some human post-vaccination sera (Huang et al., 2015, WHO, 2016a).

Analyses of the A(H1N1)pdm09 HA and NA were unable to explain the occurrence of many serious disease and several death cases, mostly in children, during 2015/2016 in Bulgaria. None of the studied viruses carried HA1 D222G/N/S or Q293H substitutions, reported to be found more frequently in patients with severe disease or fatal outcome earlier in the pandemic period (Glinsky, 2010). D222G substitution was shown to cause a shift from α2,6-sialic acid receptor specificity to mixed α2,3/α2,6-sialic acid receptor specificity, adduced thereby to facilitate lung infection (Liu et al., 2010). To investigate this further the internal genes of two Bulgarian viruses, and analogous sequences of viruses from countries that reported an increased number of clinically severe or fatal influenza cases at the beginning of the season, were analyzed. A number of changes in M1, NP, NS1 and PB2 proteins were observed in all studied subclade 6B.1 viruses but the biological significance and clinical relevance of these is unknown. However, substitutions in NP may affect a host's cytotoxic immune response because this protein contains many T-cell epitopes (Townsend and Skehel, 1984) and it has been reported that the global frequency of substitutions D2E and E125D in NS1, M105 T in NP and Q208K in M1 observed in our study has increased drastically over the last two years. (Komissarov et al., 2016). These observations indicate that the specified substitutions may provide selective advantages in virus replication or transmission.

Influenza A(H3N2) viruses played a lesser role in the 2015/2016 influenza season (10% of total detections). The small number of Bulgarian viruses sequenced belonged to subclade 3C.2a as was the case for the vast majority of A(H3N2) viruses in European countries (ECDC, 2016). While subclade 3C.2a viruses showed antigenic relatedness to the 2015/2016 vaccine virus A/Switzerland/9715293/2013 (subclade 3C.3a) (ECDC, 2016), Bulgarian and other 3C.2a viruses contained 11 HA amino acid substitutions, and six in NA, compared to the vaccine virus. Most HA substitutions were present in Bulgarian 3C.2a isolates from the previous season, with the exception of HA1 N171K and S312R, and HA2 I77V and G155E, substitutions found in viruses from the 2015/2016 season. Four of the HA substitutions were located in antigenic sites A and B. Prior studies suggested that simultaneous occurrence of at least four substitutions across two or more antigenic sites were necessary for the emergence of antigenic drift variants of epidemiological significance (Wilson and Cox, 1990, Ferguson et al., 2003, Jin et al., 2005, Eshaghi et al., 2014) and it has been reported that human antibodies appear to target mainly antigenic sites B and A, localized on the top of HA around the RBS (Chambers et al., 2015, Popova et al., 2012). Koel et al. (2013) have found that amino acid substitutions causing major antigenic changes in A(H3N2) viruses over the period 1968–2003 were located at seven positions only in HA1 in antigenic sites A and B near the RBS (Koel, 2013). In our study amino acid substitution at position 159 located in a highly exposed region of antigenic site B and the addition of a new *N*-glycosylation motif at positions 158–160 within the same antigenic site could potentially alter antigenicity. For A(H3N2) viruses a steady increase in the number of HA1 *N*-glycosylation motifs has been typical since their appearance in humans in 1968 (Blackburne et al., 2008): progenitor 1968 viruses had six HA1, one HA2, and eight NA *N*-glycosylation motifs while those in 2015/2016 had 12 or 13, one, and eight or nine respectively. These higher levels of amino acid substitution and degree of glycosylation of A/H3 viruses support their greater variability and faster evolution compared to A/H1 viruses (Eshaghi et al., 2014, Fitch et al., 1991, Wedde et al., 2015, Bedford et al., 2014). Due to the general inability of subclade 3C.2a viruses to agglutinate erythrocytes, it was not possible to assess any correlation between amino acid/glycosylation changes and antigenic properties of these viruses using HI assay.

In Bulgaria, all type B viruses detected in 2015/2016 were of the Victoria-lineage (clade 1A), a reversal compared to the three previous seasons when all belonged to the Yamagata-lineage. The Bulgarian B/Victoria-lineage viruses showed two amino acid substitutions in HA1, located in antigenic the 120-loop (positions 116–137), and 5 substitutions in NA compared to the vaccine strain B/Brisbane/60/2008 (a component of 2015/2016 quadrivalent influenza vaccines). It has been reported that the 120-loop appeared to be one of the most frequently altered regions, and amino acid substitutions in this region may strongly affect virus antigenicity (Lugovtsev et al., 2007, Wang et al., 2008, Tramuto et al., 2016). Our findings confirm previous observations that influenza B viruses evolve at a slower rate and have limited genetic diversity as compared to type A viruses (Krystal et al., 1983, Chen, 2008, Bedford et al., 2014). The results of antigenic characterization showed that the Bulgarian influenza B viruses had a close relationship with cell-culture-propagated viruses that are genetically similar to the vaccine virus. However, they were antigenically different from the hens' egg-propagated B/Brisbane/60/2008 vaccine due to acquisition of egg-adaptive amino acid substitution in HA1.

Constant monitoring of the susceptibility of influenza viruses to antivirals is necessary to allow rapid detection of viruses with reduced sensitivity. Testing of influenza viruses circulating in Bulgaria, carried out by means of genotypic and phenotypic methods, showed that all studied type A viruses were resistant to M2 blockers but both type A and type B viruses were susceptible to oseltamivir and zanamivir. In our previous study, we detected a single A(H1N1)pdm09 virus carrying NA H275Y substitution in a child treated with oseltamivir (Angelova et al., 2015). Globally, oseltamivir resistance of A(H1N1)pdm09 viruses due to NA H275Y substitution has been low (~ 1% of viruses tested) and resistance of A(H3N2) and B viruses is extremely rare (Hurt et al., 2016).

In summary, A(H1N1)pdm09 viruses of subclade 6B.1 predominated in Bulgaria during the 2015/2016 season. Despite many genetic changes, they remained antigenically closely related to the vaccine virus. In contrast, all detected type B viruses were members of the Victoria-lineage (clade 1A) differing from the B/Yamagata-lineage vaccine component, B/Phuket/3073/2013. This suggests that the 2015/2016 trivalent vaccines provided suboptimal protection against influenza B viruses. The results of this study confirm the genetic variability of circulating seasonal influenza viruses and demonstrate the need for continual antigenic and molecular surveillance for the purpose of early detection of novel genetic variants of epidemiological and clinical significance. Such information is important for the development of optimal strategies for prevention and control of influenza.

## Disclosure statement

The authors declare no conflict of interest.

## Figures and Tables

**Table 1:** Number (%) of patients infected with influenza A(H1N1)pdm09, A(H3N2) and type B viruses.

Age groups (years)	No. of patients tested	Number (%) of patients infected by influenza viruses			
		A(H1N1)pdm09	A(H3N2)	Type B	Total
0–4	367	76 (20.7)	11 (3.0)	15 (4.1)	102 (27.8)
5–14	233	32 (13.7)	9 (3.9)	32 (13.7)	73 (31.3)
15–29	133	20 (15.3)	4 (3.1)	9 (6.9)	33 (24.8)
30–64	286	65 (22.7)	4 (1.4)	14 (4.9)	83 (29.0)
≥ 65	72	14 (19.4)	2 (2.8)	2 (2.8)	18 (25.0)
Unknown	36	3 (8.3)	1 (2.8)	5 (13.9)	9 (25)
Total	1127	210 (18.6)	31 (2.8)	77 (6.8)	318 (28.2)

**Table 2:** HI antigenic analysis of influenza A(H1N1)pdm09 viruses.

Reference A(H1N1)pdm09 viruses	Genetic group	Passage history	No of isolates	Reduction in HI titre compared to homologous titre		
				≤ 2-fold	4-fold	≥ 8-fold
A/California/7/2009		E3/E2	57	57	–	–
A/Bayern/69/2009		MDCK5/MDCK1	57	52	5	–
A/Lviv/N6/2009		MDCK4/SIAT1/MDCK3	57	4	41	12
A/Christchurch/16/2010	4	E1/E3	57	–	2	55
A/Astrakhan/1/2011	5	MDCK1/MDCK5	57	57	–	–
A/St. Petersburg/27/2011	6	E1/E4	57	57	–	–
A/St. Petersburg/100/2011	7	E1/E4	57	57	–	–
A/Hong Kong/5659/2012	6А	MDCK4/MDCK2	57	57	–	–
A/South Africa/3626/2013	6B	E1/E3	57	57	–	–
A/Slovenia/2903/2015	6B.1	E4/E1	57	56	1	–
A/Israel/Q-504/2015	6B.2	*C*1/MDCK2	57	51	5	–

E — egg isolate.

**Table 3:** HI antigenic analyses of influenza B viruses (Victoria-lineage).

Reference B/Victoria-lineage viruses	Genetic group	Passage history	No of isolates	Reduction in HI titre compared to homologous titre		
				≤ 2-fold	4-fold	≥ 8-fold
B/Malaysia/2506/2004	1A	E3/E7	19	–	–	19
B/Brisbane/60/2008	1A	E4/E4	19	–	–	19
B/Malta/636714/2011	1A	E4/E1	19	–	–	19
B/South Australia/81/2012	1A	E4/E2	19	–	–	19
B/Johannesburg/3964/2012	1A	E1/E2	19	–	–	19
B/Formosa/V2367/2012	1A	MDCK1/MDCK3	19	7	12	–
B/Hong Kong/514/2009	1B	MDCK1/MDCK1	19	19	–	–
B/Ireland/3154/2016	1A	MDCK3	19	19	–	–
B/Nordrhein-Westfalen/1/2016	1A	C2/MDCK1	19	19	–	–

E — egg isolate.

**Table 4:**
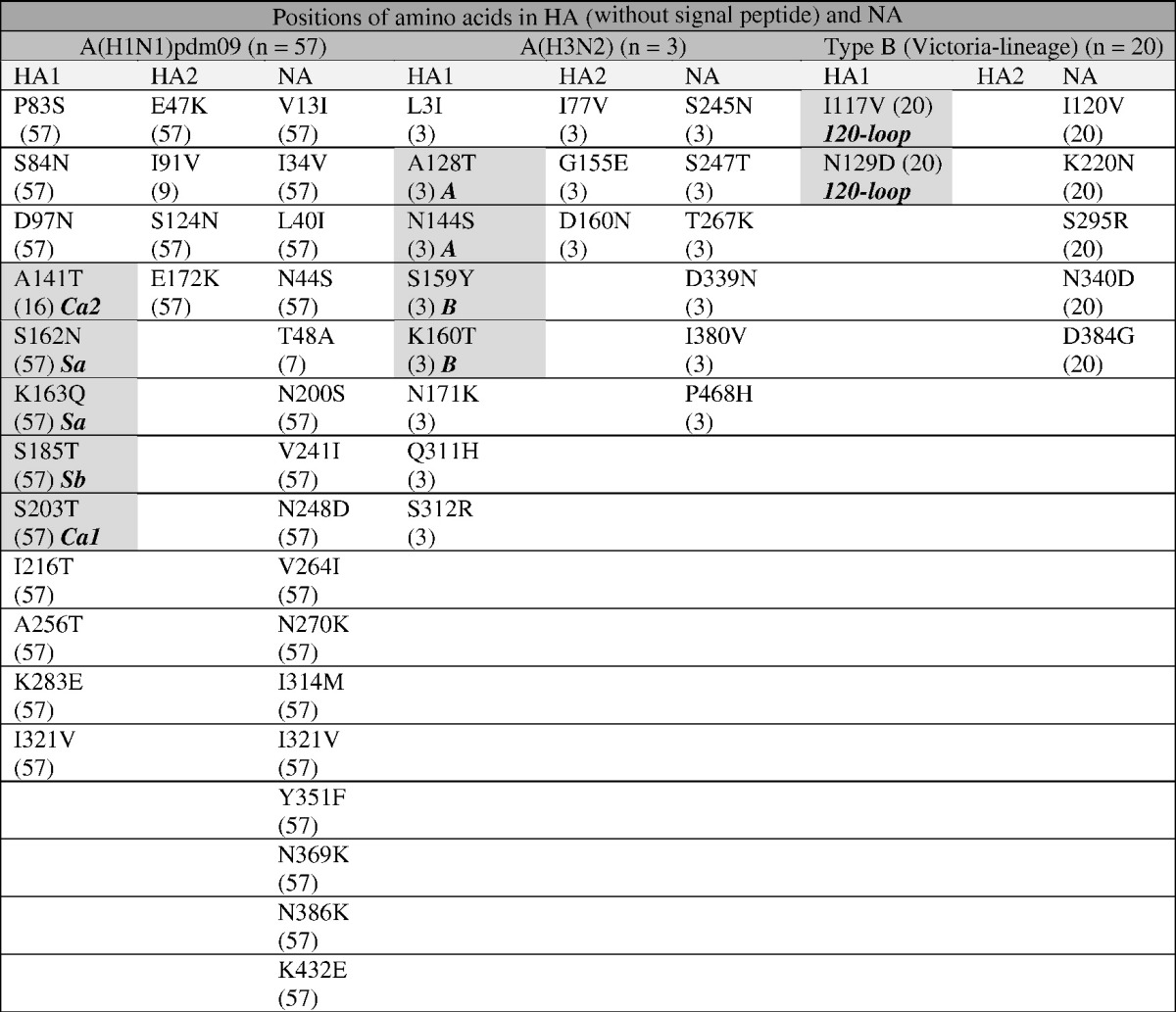
Amino acid substitutions found on the HA and NA of influenza A(H1N1)pdm09, A(H3N2) and B/Victoria-lineage viruses compared to actual vaccine viruses A/California/7/2009, A/Switzerland/9715293/2013 and B/Brisbane/60/2008, respectively.

Only amino acid substitutions found in two or more viruses are presented in this table.

The number of Bulgarian influenza viruses possessing the substitution is indicated within parentheses.

Substitutions within antigenic epitopes are highlighted in dark gray; antigenic sites are identified in bold italic.

**Table 5:** Number of positions in antigenic and receptor binding sites of HA in A(H1N1)pdm09, A(H3N2) and B/Victoria-lineage viruses with identified amino acid substitutions compared to respective vaccine viruses.

Viruses	Antigenic site					RBS
A(H1N1)pdm09	Sa	Sb	Ca1	Ca2	Cb	190 loop
	2	1	1	1	–	1
A(H3N2)	A	B	C	D	E	
	2	2	–	–	–	–
Type B	120 loop	150 loop	160 loop	190 helix		
	2	–	–	–		–

## References

[bb0005] Angelova S.G., Georgieva I.L., Teodosieva A.A., Korsun N.S. (2015). Neuraminidase inhibitor susceptibility of influenza viruses circulating in Bulgaria during the last four consecutive epidemic seasons (2011/12 to 2014/15). Am. Sci. Res. J. Eng. Technol. Sci..

[bb0010] Bautista E., Chotpitayasunondh T., Gao Z., Harper S.A., Shaw M., Uyeki T.M., Zaki S.R., Hayden F.G., Hui D.S., Kettner J.D., Kumar A., Lim M., Shindo N., Penn C., Nicholson K.G. (2010). Clinical aspects of pandemic 2009 influenza A (H1N1) virus infection. N. Engl. J. Med..

[bb0015] Beauté J., Zucs P., Korsun N., Bragstad K., Enouf V., Kossyvakis A., Griškevičius A., Olinger C.M., Meijer A., Guiomar R., Prosenc K., Staroňová E., Delgado C., Brytting M., Broberg E., European Influenza Surveillance Network (2015). Age-specific differences in influenza virus type and subtype distribution in the 2012/2013 season in 12 European countries. Epidemiol. Infect..

[bb0020] Bedford T., Suchard M.A., Lemey P., Dudas G., Gregory V., Hay A.J., McCauley J.W., Russell C.A., Smith D.J., Rambaut A. (2014). Integrating influenza antigenic dynamics with molecular evolution. Elife.

[bb0025] Blackburne B.P., Hay A.J., Goldstein R.A. (2008). Changing selective pressure during antigenic changes in human influenza H3. PLoS Pathog..

[bb0030] Chambers B.S., Parkhouse K., Ross T.M., Alby K., Hensley S.E. (2015). Identification of hemagglutinin residues responsible for H3N2 antigenic drift during the 2014–2015 influenza season. Cell Rep..

[bb0035] Chen R. (2008). The evolutionary dynamics of human influenza B virus. J. Mol. Evol..

[bb0040] Colman P.M., Hoyne P.A., Lawrence M.C. (1993). Sequence and structure alignment of paramyxovirus hemagglutinin-neuraminidase with influenza virus neuraminidase. J. Virol..

[bb0045] De Graaf M., Fouchier R.A. (2014). Role of receptor binding specificity in influenza A virus transmission and pathogenesis. EMBO J..

[bb0050] Eshaghi A., Duvvuri V.R., Li A., Patel S.N., Bastien N., Li Y., Low D.E., Gubbay J.B. (2014). Genetic characterization of seasonal influenza A (H3N2) viruses in Ontario during 2010–2011 influenza season: high prevalence of mutations at antigenic sites. Influenza Other Respir. Viruses.

[bb0055] ECDC (June 2016). Influenza Virus Characterisation Summary Europe. http://ecdc.europa.eu/en/publications/Publications/influenza-virus-characterisation-september-2016.pdf.

[bb0060] Fang Q., Gao Y., Chen M., Guo X., Yang X., Yang X., Wei L. (2014). Molecular epidemiology and evolution of A(H1N1)pdm09 and H3N2 virus during winter 2012–2013 in Beijing, China. Infect. Genet. Evol..

[bb0065] Ferguson N.M., Galvani A.P., Bush R.M. (2003). Ecological and immunological determinants of influenza evolution. Nature.

[bb0070] Fitch W.M., Leiter J.M., Li X.Q., Palese P. (1991). Positive Darwinian evolution in human influenza A viruses. Proc. Natl. Acad. Sci. U. S. A..

[bb0075] Glinsky G.V. (2010). Genomic analysis of pandemic (H1N1) 2009 reveals association of increasing disease severity with emergence of novel hemagglutinin mutations. Cell Cycle.

[bb0080] Huang K.Y., Rijal P., Schimanski L., Powell T.J., Lin T.Y., McCauley J.W., Daniels R.S., Townsend A.R. (2015). Focused antibody response to influenza linked to antigenic drift. J. Clin. Invest..

[bb0085] Horthongkham N., Athipanyasilp N., Pattama A., Kaewnapan B., Sornprasert S., Srisurapanont S. (2016). Epidemiological, clinical and virological characteristics of influenza B virus from patients at the hospital tertiary care units in Bangkok during 2011–2014. PLoS ONE.

[bb0090] Hurt A.C., Besselaar T.G., Daniels R.S., Ermetal B., Fry A., Gubareva L., Huang W., Lackenby A., Lee R.T., Lo J., Maurer-Stroh S., Nguyen H.T., Pereyaslov D., Rebelo-de-Andrade H., Siqueira M.M., Takashita E., Tashiro M., Tilmanis D., Wang D., Zhang W., Meijer A. (2016). Global update on the susceptibility of human influenza viruses to neuraminidase inhibitors, 2014–2015. Antiviral Res..

[bb0095] Ilyicheva T., Durymanov A., Susloparov I., Kolosova N., Goncharova N., Svyatchenko S. (2016). Fatal cases of seasonal influenza in Russia in 2015 ± 2016. PLoS ONE.

[bb0100] Jin H., Zhou H., Liu H., Chan W., Adhikary L., Mahmood K., Lee M.-S., Kemble G. (2005). Two residues in the hemagglutinin of A/Fujian/411/02-like influenza viruses are responsible for antigenic drift from A/Panama/2007/99. Virology..

[bb0105] Koel B.F. (2013). Substitutions near the receptor binding site determine major antigenic change during influenza virus evolution. Science.

[bb0110] Koel B.F., Mögling R., Chutinimitkul S., Fraaij P.L., Burke D.F., van der Vliet S., de Wit E., Bestebroer T.M., Rimmelzwaan G.F., Osterhaus A.D., Smith D.J., Fouchier R.A., de Graaf M. (2015). Identification of amino acid substitutions supporting antigenic change of influenza A(H1N1)pdm09 viruses. J. Virol..

[bb0115] Komissarov A., Fadeev A., Sergeeva M., Petrov S., Sintsova K., Egorova A., Pisareva M., Buzitskaya Z., Musaeva T., Danilenko D., Konovalova N., Petrova P., Stolyarov K., Smorodintseva E., Burtseva E., Krasnoslobodtsev K., Kirillova E., Karpova L., Eropkin M., Sominina A., Grudinin M. (2016). Rapid spread of influenza A(H1N1)pdm09 viruses with a new set of specific mutations in the internal genes in the beginning of 2015/2016 epidemic season in Moscow and Saint Petersburg (Russian Federation). Influenza Other Respir. Viruses.

[bb0120] Korsun N., Angelova S., Georgieva I. (2015). Influenza virus activity during the 2013/2014 and 2014/2015 seasons in Bulgaria. C. R. Acad. Bulg. Sci..

[bb0125] Krystal M., Young J.F., Palese P., Wilson I.A., Skehel J.J. (1983). Sequential mutations in hemagglutinins of influenza B virus isolates: definition of antigenic domains. Proc. Natl. Acad. Sci. U. S. A..

[bb0130] Laplante J., George K.St. (2014). Antiviral resistance in influenza viruses. Laboratory testing. Clin. Lab. Med..

[bb0135] Lee H.K., Tang J.W.-T., Kong D.H.-L., Loh T.P., Chiang D.K.-L. (2013). Comparison of mutation patterns in full-genome A/H3N2 influenza sequences obtained directly from clinical samples and the same samples after a single MDCK passage. PLoS ONE.

[bb0140] Liu Y., Childs R.A., Matrosovich T., Wharton S., Palma A.S. (2010). Altered receptor specificity and cell tropism of D222G hemagglutinin mutants isolated from fatal cases of pandemic A(H1N1) 2009 influenza virus. J. Virol..

[bb0145] Lugovtsev V.Y., Vodeiko G.M., Strupczewski C.M., Ye Z., Levandowski R.A. (2007). Generation of the influenza B viruses with improved growth phenotype by substitution of specific amino acids of hemagglutinin. Virology.

[bb0150] Matrosovich M., Matrosovich T., Carr J., Roberts N.A., Klenk H.D. (2003). Over expression of the a-2,6-sialyltransferase in MDCK cells increases influenza virus sensitivity to neuraminidase inhibitors. J. Virol..

[bb0155] Neumann N., Noda T., Kawaoka Y. (2009). Emergence and pandemic potential of swine-origin H1N1 influenza virus. Nature.

[bb0160] Popova L., Smith K., West A.H., Wilson P.C., James J.A. (2012). Immunodominance of antigenic site B over site A of hemagglutinin of recent H3N2 influenza viruses. PLoS ONE.

[bb0165] Ramadhany R., Yasugi M., Nakamura S., Daidoji T., Watanabe Y., Takahashi K., Ikuta K., Nakaya T. (2012). Tropism of pandemic 2009 H1N1 influenza A virus. Front. Microbiol..

[bb0170] Skehel J.J., Stevens D.J., Daniels R.S., Douglas A.R., Knossow M., Wilson I.A., Wiley D.C. (1984). A carbohydrate side chain on hemagglutinins of Hong Kong influenza viruses inhibits recognition by a monoclonal antibody. Proc. Natl. Acad. Sci. USA.

[bb0175] Sriwilaijaroen N., Suzuki Y. (2012). Molecular basis of the structure and function of H1 hemagglutinin of influenza virus. Proc. Jpn. Acad. Ser. B. Phys. Biol. Sci..

[bb0180] Townsend A.R., Skehel J.J. (1984). The influenza A virus nucleoprotein gene controls the induction of both subtype specific and cross-reactive cytotoxic T cells. J. Exp. Med..

[bb0185] Tramuto F., Orsi A., Maida C.M., Costantino C., Trucchi C., Alicino C., Vitale F., Ansaldi F. (2016). The molecular epidemiology and evolutionary dynamics of influenza B virus in two Italian regions during 2010–2015: the experience of Sicily and Liguria. Int. J. Mol. Sci..

[bb0190] Wang Q., Cheng F., Lu M., Tian X., Ma J. (2008). Crystal structure of unliganded influenza B virus hemagglutinin. J. Virol..

[bb0195] Wedde M., Biere B., Wolff T., Schweiger B. (2015). Evolution of the hemagglutinin expressed by human influenza A(H1N1)pdm09 and A(H3N2) viruses circulating between 2008–2009 and 2013–2014 in Germany. Int. J. Med. Microbiol..

[bb0200] WHO (2011). Manual for the Laboratory Diagnosis and Virological Surveillance of Influenza. http://whqlibdoc.who.int/publications/2011/9789241548090_eng.pdf.

[bb0205] WHO (2014). Influenza (Seasonal) Fact Sheet No. 211. http://www.who.int/mediacentre/factsheets/fs211/en/.

[bb0210] WHO (2016). Recommended Composition of Influenza Virus Vaccines for Use in the 2017 Southern Hemisphere Influenza Season. http://www.who.int/influenza/vaccines/virus/recommendations/201609_recommendation.pdf?ua=1.

[bb0215] WHO (2016). Risk Assessment of the 2015–2016 Influenza Season in the WHO European Region, Week 40/2015 to Week 04/2016. http://www.euro.who.int/__data/assets/pdf_file/0011/301115/Risk-assessment-influenza-season-week40-15-to-week04-16.pdf.

[bb0220] WHO (2016). Summary of Neuraminidase Amino Acid Substitutions Associated with Reduced Inhibition by Neuraminidase Inhibitors (NAI). http://www.who.int/influenza/gisrs_laboratory/antiviral_susceptibility/nai_overview/en/.

[bb0225] WHO (2016). Worldwide Influenza Centre, London. September 2016 Interim Report. Report Prepared for the WHO Annual Consultation on the Composition of Influenza Vaccine for the Southern Hemisphere 2017. 26th–28th September 2016. https://crick.ac.uk/media/326439/september_2016_interim_report.pdf.

[bb0230] Wiley D.C., Skehel J.J. (1987). The structure and function of the hemagglutinin membrane glycoprotein of influenza virus. Annu. Rev. Biochem..

[bb0235] Wilson I.A., Cox N. (1990). Structural basis of immune recognition of influenza virus hemagglutinin. Annu Rev. Immunol.

[bb0240] Yang H., Carney P., Stevens J. (2010). Structure and receptor binding properties of a pandemic H1N1 virus hemagglutinin. Version 2. PloS Curr..

